# The impact of teacher feedback on medical students’ self-regulated learning: a serial mediation model of teacher-student interaction and sense of school belonging

**DOI:** 10.1186/s12909-025-06888-3

**Published:** 2025-02-25

**Authors:** Jing Tian, Zhidan Hui, Hongde Lei

**Affiliations:** School of Education, Huazhong University of Science and Technology, 1037 Luoyu Road, Wuhan, 430074 China

**Keywords:** Medical students, Teacher feedback, Self-regulated learning, Teacher-student interaction, Sense of school belonging

## Abstract

**Background:**

Self-regulated learning is crucial for medical students because of the rigorous and dynamic nature of medical education. Previous studies have demonstrated the positive influence of teacher feedback on students’ self-regulated learning, particularly in basic education contexts. However, research exploring the mechanisms through which teacher feedback affects self-regulated learning among medical students in higher education contexts remains limited. This study aims to investigate the impact of teacher feedback on medical students’ self-regulated learning, with a focus on the mediation roles of teacher-student interaction and sense of school belonging.

**Methods:**

Data were collected from a comprehensive survey of undergraduates at H University, a prestigious research-oriented institution in China. The sample included 6,546 medical students spanning five academic years (2014, 2016, 2018, 2021, and 2023). The Student Survey of Learning and Development questionnaire was utilized to collect data, and teacher feedback, teacher-student interaction, sense of school belonging, and self-regulated learning were chosen as variables in this study. Structural equation modeling was employed to analyze the relationships among these variables, with mediation and serial mediation effects assessed via the bootstrap method.

**Results:**

Teacher feedback significantly predicted medical students’ self-regulated learning (*β* = 0.136, *p* < 0.001). Teacher-student interaction (*β* = 0.377, *p* < 0.001) and sense of school belonging (*β* = 0.325, *p* < 0.001) were found to mediate the relationship between teacher feedback and self-regulated learning. The mediation effects, with effect sizes of 0.116 for teacher-student interaction and 0.045 for sense of school belonging, accounted for 37.91% and 14.71% of the total effect, respectively. Additionally, there was a serial mediation model between teacher feedback and self-regulated learning that had a value of 0.009, accounting for 2.94% of the total effect.

**Conclusion:**

This study provides empirical evidence supporting the role of teacher feedback in promoting medical students’ self-regulated learning, with teacher-student interaction and sense of school belonging serving as important mediating factors. These findings have implications for designing effective educational interventions to cultivate self-regulated learners capable of adapting to the rapidly evolving landscape of medical knowledge and practice.

## Introduction

Self-regulated learning is not only a matter of individual freedom in decision-making but also a crucial capability for deep learning [1]. It encompasses the process of learners setting their own learning objectives, choosing their own methods, monitoring their progress, and evaluating their outcomes [2]. Research has shown that self-regulated learning significantly enhances students’ engagement [3] and directly impacts their academic performance [4]. For medical students, self-regulated learning is particularly important because medicine is a type of “hard science” characterized by rapid knowledge updates, heavy learning tasks, high challenges and long durations, which require learners to have stronger self-regulated learning ability. This ability not only directly affects the learning of medical students during their school years but also has a long-term impact on their career after graduation [5, 6]. If medical students can effectively engage in self-regulated learning, then they will be better equipped to adapt to the continuous evolution of medical knowledge and the dynamic nature of their future careers. Therefore, medical schools generally attach great importance to fostering students’ self-regulated learning to meet the demands of lifelong learning and address the challenges of career advancement [7].

It is crucial to identify the factors that influence students’ self-regulated learning to cultivate this ability.The multifaceted nature of these factors has been emphasized by social cognitive theory, with environmental aspects, particularly teachers, playing a pivotal role[5]. As a result, a close relationship between teacher feedback and students’self-regulated learning has been revealed by some studies [8, 9], especially in basic education contexts [10]. Although some previous studies have delved into the impact of teacher feedback on students’ self-regulated learning, this topic requires further research on medical students in higher education contexts. Medical students face heavy academic pressure and have a strong demand for teacher feedback because they urgently need teachers to provide academic guidance and emotional support [11]. Some relevant studies have explored the mechanisms through which the integration of erroneous examples and elaborate feedback can influence the learning process, especially within the context of acquiring complex diagnostic knowledge [12, 13]. Moreover, Kopp et al. confirmed that students who received elaborate feedback rated their learning outcomes significantly higher than those who received knowledge of correct result feedback alone [14]. However, the research has not yet elucidated the specific mechanisms through which teacher feedback influences medical students’ self-regulated learning, nor has it identified effective strategies from the teacher’s perspective on how to promote such learning. This research gap is detrimental to the learning and development of medical students, thereby hindering the fulfillment of the pressing need for high-quality physicians who can uphold public health and human well-being. Therefore, it is imperative to identify teacher feedback as a pivotal factor and meticulously investigate its influence on medical students’ self-regulated learning, along with the mediation processes that underlie this relationship. Such an endeavor will not only deepen the understanding of self-regulated learning but also help to implement practical strategies for developing more well-educated healthcare professionals.

In summary, this study aims to gain deeper insights into how teacher feedback can effectively promote medical students’ self-regulated learning. To achieve this goal, it utilizes data from a learning and development survey conducted specifically among undergraduates at a prominent university in China. The study focuses on a sample of medical students and examines the relationship between teacher feedback and self-regulated learning. Drawing on Bronfenbrenner’s ecological systems theory, this study provides a comprehensive framework for understanding how individuals develop within diverse environmental systems [15]. The theory posits that an individual’s development is influenced by the intricate interplay of various environmental systems, including microsystems (e.g., family, peers), mesosystems (e.g., school, community), exosystems (e.g., cultural norms, societal values), and macrosystems (e.g., political, economic structures). It has been widely used in social work, psychology, and education to analyze the complex relationships between individuals and their environments [16–19]. Within the educational context, Bronfenbrenner’s ecological systems theory has been applied to explore factors that contribute to students’ academic development, including teacher-student interaction and students’ sense of belonging [20, 21]. Drawing on this theoretical framework, the current study examines how teacher feedback, teacher-student interaction, and sense of school belonging jointly influence medical students’ self-regulated learning.

### The relationship between teacher feedback and self-regulated learning

Self-regulated learning is a multifaceted construct that is influenced by personal traits, social environments, and specific learning contexts [22]. The educational institution, particularly in higher education contexts, serves as the primary formal learning environment and is instrumental in fostering students’ self-regulated learning [23]. Within this academic setting, the relationship between teacher feedback and students’ self-regulated learning has garnered substantial attention [8]. Several recent studies have underscored the pivotal role of teacher feedback in promoting self-regulated learning among college students. Haimovitz and Henderlong demonstrated that positive feedback from teachers can significantly enhance college students’ intrinsic motivation and perceived competence [24]. Moreover, students’ appreciation of such feedback fosters their self-efficacy, ultimately encouraging deeper engagement in the learning process. This finding is echoed by Zheng et al., who categorized teacher feedback into various types, such as validating, guiding, scaffolding, praise, and criticism, and found significant positive correlations between these feedback types and various dimensions of online self-directed learning among college students [25]. In addition, recent research has highlighted the importance of considering the quality and timing of teacher feedback. For example, Chen examined the effect of feedback timing on college students’ self-regulated learning and reported that timely feedback helps students better adjust their learning strategies and improve their learning efficiency and participation [26]. Similarly, Hattie and Timperley emphasized the power of feedback in guiding students’ learning and encouraging them to take responsibility for their own progress [27]. Drawing from these insights, the current study proposes Hypothesis 1: Teacher feedback can predict medical students’ self-regulated learning. This hypothesis is grounded in the literature, which consistently demonstrates that teacher feedback plays a vital role in fostering students’ self-regulated learning, particularly in higher education contexts.

### The mediation role of teacher-student interaction

Teacher-student interaction is a multifaceted form of engagement that integrates emotional, cognitive, and behavioral aspects of communication, representing a comprehensive range of educational activities and interactions [28]. This holistic approach to interaction is crucial for fostering an effective learning environment. A study by Kwok et al. in 2022 showed that teacher-student interaction is one of the key factors in stimulating students’ motivational regulation strategies [29], which can thereby enhance their self-regulated learning. Choi and Won demonstrated in their study of 163 nursing students that teacher-student interaction positively promotes self-regulated learning [30]. In effective teacher-student interaction, students feel the teachers’ attention and encouragement, which fosters motivation and willingness to engage in learning, thus increasing their level of self-regulated learning [31].

Teacher feedback can be categorized as positive or negative, with varying degrees of effectiveness. Zacharias discovered through a comprehensive analysis of both quantitative and qualitative data that students favor specific teacher feedback, appreciating its practical utility in the learning process [32]. Effective teacher feedback entails identifying students’ strengths and areas needing development, as well as offering specific educational advice and the necessary emotional support and encouragement. A round-table discussion with 28 teachers revealed that students generally crave recognition and praise from teachers, which contributes to harmonious teacher-student interactions [33]. On the basis of these findings, this paper proposes Hypothesis 2: Teacher-student interaction mediates the relationship between teacher feedback and medical students’ self-regulated learning.

### The mediation role of sense of school belonging

The sense of school belonging is a profound experience in which students feel accepted, respected, recognized, and supported within the school environment, which is crucial for their holistic development [34]. Research has demonstrated that a strong sense of school belonging significantly influences students’ performance and well-being, enhancing not only their academic achievements but also their prosocial behavior and psychological health [35, 36]. Won et al. further established that this sense of belonging is crucial for students’ academic development, as it underpins effective time management, sustains a constructive learning attitude, and stimulates active participation in educational activities [37]. In fact, when students experience a strong sense of school belonging, they internalize this feeling as motivation, externalize it as proactive learning behaviors, and integrate it into their daily studies, thereby improving their self-regulated learning [38].

According to Bandura’s triadic reciprocal determinism theory, there is a close interplay among an individual’s cognitive, environmental, and behavioral factors [39]. Within this theoretical framework, sense of school belonging acts as a bridge between environmental and behavioral factors, playing a vital role. Teacher feedback, as an environmental element, influences students’ perceptions of their academic environment. A mixed-methods study by Atlas showed that teacher feedback can help students enhance their identification with the school’s physical conditions and cultural atmosphere, thereby increasing their sense of belonging [40]. On the basis of these findings, this paper proposes Hypothesis 3: Sense of school belonging mediates the relationship between teacher feedback and medical students’ self-regulated learning.

### The serial mediation roles of teacher-student interaction and sense of school belonging

Social identity theory posits that humans construct their social identities through the sense of belonging to various social groups. When students interact with teachers in school settings and experience positive communication and emotional support, their sense of school belonging is enhanced [41]. For example, Ahmadi et al. surveyed 25,000 high school students and constructed a hierarchical linear model in 2020, finding that a sense of fairness at the school level and teacher-student relationships can explain variations in students’ sense of school belonging [42]. Additionally, a literature review of 32 studies on the relationship between teacher-student interaction and sense of belonging revealed that in positive teacher-student interaction, students are more likely to perceive fairness and justice in school life, receive encouragement and support to participate in various activities, and gain growth experiences from these engagements [43]. Another study involving interviews with 9 high school teachers revealed that students with mental health and social isolation issues can establish a deep understanding of and trust with teachers only through frequent and positive interactions, laying the foundation for harmonious school life [44]. Although some previous studies have delved into the relationship between teacher-student interaction and sense of school belonging in secondary and high schools, this topic still requires further research in the field of medical education. On the basis of these findings, this paper proposes Hypothesis 4: Teacher-student interaction and sense of belonging jointly mediate the relationship between teacher feedback and medical students’ self-regulated learning.

**Fig. 1:**
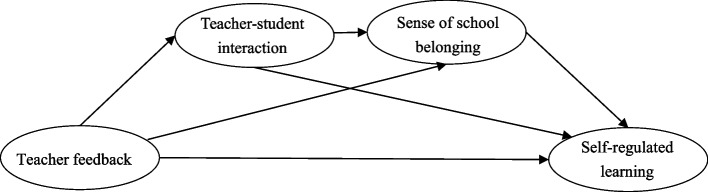
Conceptual framework

In summary, on the basis of the four hypotheses mentioned above, this paper proposes a conceptual framework (Fig. 1) illustrating the mechanism by which teacher feedback influences the self-regulated learning of medical students.

## Methods

The participants in this study were from H University, a prestigious research-oriented institution situated in China. Renowned as the “epitome of higher education in New China,” H University serves as an emblematic entity for the present analysis [45]. Furthermore, the medical college at this university boasts a prolonged history and stands as one of the foundational incubators of modern medical education in China. Given its stringent admission criteria and academic prowess, the medical college typically attracts high-achieving students. To gain insights into and foster student learning and development, H University has conducted a series of five surveys over the years 2014, 2016, 2018, 2021, and 2023. A comprehensive dataset comprising 6,546 valid questionnaires was obtained from medical students as follows: 1,504 students from Grade 1, 1,569 students from Grade 2, 1,559 students from Grade 3, 1,099 students from Grade 4, and 815 students from Grade 5.

### Measures and instruments

The primary measurement instrument utilized in this study is the Student Survey of Learning and Development (SSLD) questionnaire, which originated from H University. This questionnaire has undergone rigorous testing and has demonstrated robust reliability and validity [46]. It is a comprehensive self-report tool encompassing five sections, namely, academic expectancy, school support, assessment and feedback, student engagement, and learning outcomes, with a total of 21 indicators [46].

In May 2014, the first survey was administered to all undergraduate students, yielding a response rate of 37.66% (12,134 out of 32,220) and a valid return rate of 93.09% (11,295 out of 12,134) [47]. The internal consistency reliability coefficients for the SSLD indicators were high, with five coefficients ranging from 0.7 to 0.8, twelve coefficients ranging from 0.8 to 0.9, and four coefficients exceeding 0.9 [46]. These coefficients align with psychometric standards, indicating a high level of reliability. Exploratory factor analysis further confirmed the good internal structural validity of the SSLD questionnaire, and correlation analysis demonstrated its satisfactory criterion-related validity.

For the purposes of this study, the following specific indicators were selected: teacher feedback, teacher-student interaction, sense of school belonging, and self-regulated learning. The reliability coefficients for these indicators were 0.773, 0.930, 0.809, and 0.868, respectively. A comprehensive analysis was conducted on these four variables across five distinct cohorts of medical students, spanning the years 2014, 2016, 2018, 2021, and 2023. Following exploratory factor analysis of the integrated data, the Cronbach’s α coefficient for the overall items of the four variables across the five rounds of questionnaires was 0.915, indicating high internal consistency. The details of each variable in this study are outlined as follows:

#### Teacher feedback

The teacher feedback scale used in this study comprises five items, which assess various aspects such as “Feedback provided by teachers after classroom tests,” “Feedback following individual or group class presentations,” and “Feedback after course homework.” Responses to these items are rated on a four-point Likert scale ranging from 1 (“Very Untimely”) to 4 (“Very Timely”). The Cronbach’s α coefficient for the teacher feedback items is 0.898, indicating strong reliability. The results of the confirmatory factor analysis further validated the structural integrity of the scale. Specifically, the Goodness of Fit Index (GFI) was 0.998, the Adjusted Goodness of Fit Index (AGFI) was 0.982, the Comparative Fit Index (CFI) was 0.998, the Tucker-Lewis Index (TLI) was 0.990, and the Root Mean Square Error of Approximation (RMSEA) was 0.054. These indices collectively suggest excellent structural validity, thereby reinforcing the robustness of the teacher feedback scale used in this study.

#### Teacher-student interaction

The teacher-student interaction scale used in this study encompasses five items, which assess diverse aspects such as “Communicating about course content with teachers,” “Discussing course-related queries with teachers,” and “Sharing emotional problems with teachers.” Responses to these items are rated on a four-point Likert scale ranging from 1 (“Never”) to 4 (“Very often”), where higher scores signify more frequent teacher-student interactions experienced by medical students. The Cronbach’s α coefficient for the teacher-student interaction items is 0.906, indicating a high degree of reliability. The results of the confirmatory factor analysis further support the structural validity of the scale. Specifically, the GFI was 0.999, the AGFI was 0.986, the CFI was 0.998, the TLI was 0.998, and the RMSEA was 0.023. These indices collectively suggest an excellent fit of the model to the data, thereby reinforcing the structural validity of the teacher-student interaction scale used in this study.

#### Sense of school belonging

Sense of school belonging scale used in this study includes four items that assess aspects such as “The school values undergraduate education” and “The school values students’ opinions.” The responses are rated on a four-point Likert scale ranging from 1 (“Strongly Disagree”) to 4 (“Strongly Agree”), with higher scores indicating a stronger sense of school belonging among medical students. The Cronbach’s α coefficient for sense of school belonging items is 0.860, which reflects good reliability. The results of the confirmatory factor analysis are GFI = 0.999, AGFI = 0.992, CFI = 0.999, TLI = 0.995, and RMSEA = 0.038, suggesting good structural validity.

#### Self-regulated learning

Self-regulated learning scale used in this study encompasses nine items that assess aspects such as “I have a clear development plan for my future,” “I often analyze and improve my learning methods,” and “Even when learning tasks are boring or monotonous, I can persist in completing them.” The responses are rated on a four-point Likert scale ranging from 1 (“Strongly Disagree”) to 4 (“Strongly Agree”), with higher scores indicating a higher level of self-regulated learning ability among medical students. The Cronbach’s α coefficient for the self-regulated learning items is 0.894, indicating good reliability. The results of the confirmatory factor analysis are GFI = 0.991, AGFI = 0.979, CFI = 0.991, TLI = 0.985, and RMSEA = 0.047, suggesting good structural validity.

### Data analysis

This study applied SPSS 26.0 and AMOS 26.0 for data analysis, following these sequential steps. First, Harman’s single-factor test was used to check for common method bias. Second, correlation analysis was used to examine the relationships among four variables: teacher feedback, teacher-student interaction, sense of school belonging, and self-regulated learning. Control variables were determined via independent samples t tests and one-way ANOVA. Structural equation modeling was used to analyze the interrelations among the key variables further. Finally, mediation and serial mediation effects were assessed via the bootstrap method with 5,000 resamples.

## Results

### Common method bias test

This study utilized Harma’s single-factor test to assess common method bias. The test resulted in four eigenvalues exceeding one, with the primary factor explaining 36.328% of the total variance. This figure which falls short of the 40% benchmark suggests that common method bias does not significantly affect the research findings [48].

### Descriptive statistics and correlation analysis results

Descriptive statistics and correlation analyses were performed for the variables in this study, as presented in Table 1. The correlations among teacher feedback, teacher-student interaction, sense of school belonging, and self-regulated learning were statistically significant. This suggests that the variables are suitable for further mediation analysis.

**Table 1:** Descriptive statistics and correlation analysis results

Variables	*M*	*SD*	1	2	3	4
1 Teacher feedback	3.035	0.621	**1**			
2 Teacher-student interaction	2.193	0.767	0.311^**^	**1**		
3 Sense of school belonging	3.339	0.599	0.409^**^	0.314^**^	**1**	
4 Self-regulated learning	2.910	0.540	0.330^**^	0.524^**^	0.346^**^	**1**

*N* = 6546. p^*^ < 0.05, ^**^p < 0.01, ^***^p < 0.001 (two-tailed tests)

### Contrasts in traits and multi-variable assessments of participants

Table 2 presents the descriptive associations between participants’ characteristics and their scores in terms of teacher feedback, teacher-student interaction, sense of school belonging, and self-regulated learning. The table also reveals notable gender-based disparities in several domains: teacher feedback (*t* = 2.483, *p* < 0.05), sense of school belonging (*t* = 5.871, *p* < 0.001), and self-regulated learning (*t* = 3.928, *p* < 0.001). Furthermore, grade level was also found to be a significant factor influencing teacher feedback (*F* = 17.102, *p* < 0.001), sense of school belonging (*F* = 6.926, *p* < 0.001), teacher-student interaction (*F* = 7.803, *p* < 0.001), and self-regulated learning (*F* = 2.725, *p* < 0.05). Additionally, the year of the survey emerged as a significant predictor of differences in various aspects, including teacher feedback (*F* = 567.675, *p* < 0.001), sense of school belonging (*F* = 86.495, *p* < 0.001), teacher-student interaction (*F* = 80.164, *p* < 0.001), and self-regulated learning (*F* = 36.203, *p* < 0.001).

**Table 2:** Variance analysis and description of each variable

Variables		*N* (%)	**Teacher feedback**	**Teacher‒student interaction**	**Sense of school belonging**	**Self-regulated learning**
			*M* ± *SD*	*M* ± *SD*	*M* ± *SD*	*M* ± *SD*
Gender	Female	3613(55.2)	3.052 ± 0.609	2.184 ± 0.754	3.379 ± 0.575	2.934 ± 0.530
	Male	2933(44.8)	3.014 ± 0.610	2.204 ± 0.784	3.292 ± 0.625	2.881 ± 0.553
	*t/F*		2.483^*^	−1.004	5.871^***^	3.928^***^
	*P*		0.013	0.315	0.000	0.000
Grade	One	1504(23.0)	3.095 ± 0.580	2.171 ± 0.759	3.384 ± 0.600	2.922 ± 0.543
	Two	1569(24.0)	3.103 ± 0.590	2.174 ± 0.762	3.379 ± 0.579	2.916 ± 0.560
	Three	1559(23.8)	2.958 ± 0.658	2.144 ± 0.783	3.289 ± 0.616	2.873 ± 0.526
	Four	1099(16.8)	2.974 ± 0.649	2.245 ± 0.756	3.299 ± 0.599	2.925 ± 0.539
	Five	815(12.5)	3.027 ± 0.618	2.294 ± 0.765	3.354 ± 0.598	2.935 ± 0.525
	*t/F*		17.102^***^	6.926^**^	7.803^***^	2.725^*^
	*P*		0.000	0.001	0.000	0.028
Year of survey	2014	1473(22.5)	2.451 ± 0.535	1.960 ± 0.722	3.102 ± 0.674	2.807 ± 0.503
	2016	1541(23.5)	3.204 ± 0.569	2.318 ± 0.798	3.410 ± 0.565	2.996 ± 0.568
	2018	1674(25.6)	3.192 ± 0.503	2.142 ± 0.720	3.404 ± 0.543	2.881 ± 0.531
	2021	904(13.8)	3.184 ± 0.542	2.140 ± 0.795	3.370 ± 0.587	2.870 ± 0.536
	2023	954(14.6)	3.247 ± 0.526	2.487 ± 0.701	3.448 ± 0.536	3.020 ± 0.532
	*t/F*		567.675^***^	88.495^***^	80.164^***^	36.203^***^
	*P*		0.000	0.000	0.000	0.000

*N* = 6546. ^*^p < .05, ^**^p < .01, ^***^p < .001 (two-tailed tests)

### Construction of structural equation model and mediation analysis

#### Structural equation model indicators

This study investigated the mediation effects of teacher-student interaction and sense of school belonging on the relationship between teacher feedback and self-regulated learning by constructing a structural equation model. Following a meticulous review of the modification indices [49], potential avenues for enhancing the model were identified. This process involves scrutinizing the residuals and assessing whether the incorporation or modification of pathways between variables could improve model fit without leading to overfitting. Through this iterative approach, the model was refined to ensure that each estimated parameter was both theoretically grounded and statistically significant, thereby optimizing the overall model fit. The refined model demonstrated the following fit indices: χ^2^ = 24.952 with 11 degrees of freedom (df), GFI = 0.999, AGFI = 0.997, CFI = 0.999, TLI = 0.999, RMSEA = 0.014, and SRMR = 0.0046. The χ^2^/df ratio fell within the acceptable range, and all other indices met the established standards in the field [50], indicating a well-fitting model.

#### Structural equation model for the serial mediation effects

**Fig. 2:**
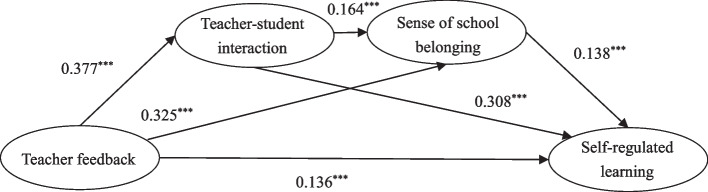
Serial mediation model (^***^ *p* < 0.001)

In subsequent analyses, gender, grade, and year of survey were controlled for in the mediation analysis. The results of the structural equation modeling are depicted in Fig. 2. Teacher feedback positively predicts self-regulated learning (*β* = 0.136, *p* < 0.001), teacher-student interaction (*β* = 0.377, *p* < 0.001), and sense of school belonging (*β* = 0.325, *p* < 0.001). Teacher-student interaction positively predicts sense of school belonging (*β* = 0.164, *p* < 0.001) and self-regulated learning (*β* = 0.308, *p* < 0.001). Sense of school belonging also positively predicts self-regulated learning (*β* = 0.138, *p* < 0.001).

#### Results of the mediation effect test

The results of the mediation analysis are presented in Table 3. The 95% confidence intervals for the mediation effects of teacher-student interaction and sense of school belonging between teacher feedback and medical students’ self-regulated learning are [0.105, 0.128] and [0.037, 0.053], respectively. Since both intervals do not include zero, they suggest that the mediation effects are statistically significant. The mediation effects, with effect sizes of 0.116 for teacher-student interaction and 0.045 for sense of school belonging, account for 37.91% and 14.71% of the total effect, respectively, indicating that both factors significantly contribute to the partial mediation of the relationship between teacher feedback and self-regulated learning.

**Table 3:** Analysis of the mediation effects of teacher feedback on self-regulated learning

Path	Effect value	Effect size	SE	95% CI
Total effect	0.306	100.00%	0.011	[0.284, 0.327]
Direct effect	0.136	44.44%	0.010	[0.115, 0.156]
Indirect effect	0.170	55.56%	0.007	[0.155, 0.184]
1.TF—TI—SL	0.116	37.91%	0.006	[0.105, 0.128]
2.TF—SSB—SL	0.045	14.71%	0.004	[0.037, 0.053]
3.TF—TI—SSB—SL	0.009	2.94%	0.001	[0.007, 0.010]

*TF* teacher feedback, *TI* teacher-student interaction, *SSB* sense of school belonging, *SL* self-regulated learning

Furthermore, the serial mediation effects of teacher-student interaction and sense of school belonging between teacher feedback and medical students’ self-regulated learning have a 95% confidence interval of [0.007, 0.010]. The confidence interval excluding zero demonstrates that the serial mediation effects are statistically significant, with an effect size of 0.009, constituting 2.94% of the overall impact.

## Discussion and conclusion

This study explores the relationship between teacher feedback and medical students’ self-regulated learning and examines the mediation roles of teacher-student interaction and sense of school belonging. The results of this study provides a deeper understanding of how teacher feedback is associated with the self-regulated learning of medical students, yielding practical implications for enhancing their self-regulated learning.

### Teacher feedback predicts self-regulated learning

This study confirms that teacher feedback is a significant and positive predictor of the self-regulated learning of medical students, supporting Hypothesis 1. This result implies that medical students who receive frequent and prompt feedback are more likely to engage in self-regulated learning. Interestingly, this outcome differs from those of several prior studies, including a study by Guo in 2020, which reported no link between teacher feedback and self-regulated learning in first-year high school students [51]. The inconsistency might stem from variations in the survey tools employed across studies and the distinct learning strategies typical of high school versus undergraduate students. In the context of medical education, constructive and timely feedback, along with clear expectations from and supportive communication with teachers, can significantly increase students’ self-regulated learning [52].

Self-determination theory asserts that social environments foster human motivation by fulfilling three fundamental psychological needs: autonomy, competence, and relatedness [53, 54]. In the context of educational practice, teacher feedback, a vital component of the academic environment, has the potential to bolster medical students’ cognitive, emotional, and behavioral engagement by addressing these essential psychological needs [55]. Specifically, timely feedback from teachers is crucial for medical students, as it amplifies their sense of competence and nurtures both emotional well-being and cognitive engagement, which are indispensable for effective learning outcomes [56]. This feedback not only acknowledges students’ efforts but also serves as a motivational catalyst, encouraging them to persist in their academic endeavors and thereby enriching their educational experiences [57]. Conversely, medical students who do not receive timely feedback may experience feelings of frustration and a lack of appreciation, which may adversely impact their motivation and self-efficacy [58]. In summary, positive and prompt teacher feedback can foster a supportive learning environment that makes medical students feel understood and supported, ultimately enhancing their self-regulated learning.

### The mediation role of teacher-student interaction

This study demonstrates a significant mediation effect of teacher-student interaction between teacher feedback and the self-regulated learning of medical students, confirming Hypothesis 2. This suggests that teacher feedback can indirectly enhance medical students’ self-regulated learning by stimulating positive interactions between students and teachers [32]. When teachers approach the educational tasks and assessments of medical students with diligence and responsibility and provide timely feedback, students are more likely to understand teachers’ expectations and establish positive interactive relationships with teachers. Such interactions simultaneously assist medical students in clarifying their learning objectives and stimulate their motivation to learn, thereby enhancing their self-regulated learning [31].

The research findings align with Skinner’s reinforcement theory [59], indicating that teacher feedback functions as a “stimulus” that elicits a “response” from medical students, particularly in terms of the frequency and depth of teacher-student interaction. Specifically, when medical students receive prompt feedback, their willingness and motivation to engage with teachers are enhanced, thereby fostering increased interactions [60]. These interactions facilitate the acquisition of academic assistance and guidance while making students feel cared for and supported by their teachers. Consequently, this support increases students’ confidence and self-efficacy [10]. Furthermore, medical students who frequently receive teacher feedback are more inclined to establish trust with their teachers, which encourages more proactive communication. This positive interactive relationship can reinforce the motivation of medical students to learn. Moreover, guidance from teachers can assist students in discovering suitable learning methods [61]. As a result, students are more likely to actively set study plans, engage in classroom discussions, complete assignments, and cultivate self-regulated learning as a habit.

### The mediation role of sense of school belonging

This study reveals that the mediation effect of sense of school belonging on the relationship between teacher feedback and medical students’ self-regulated learning is significant, validating Hypothesis 3. These findings suggest that teacher feedback can indirectly enhance the self-regulated learning of medical students by strengthening their sense of school belonging. This finding aligns with prior studie, indicating that when teachers actively acknowledge students’ strengths, encourage improvement, and offer timely feedback, medical students are more likely to feel supported and accepted, thus fostering a strong sense of belonging at school [40, 62] This sense of belonging can enhance students’ positive emotions and participation, thereby promoting their active engagement in learning activities and improving their self-regulated learning [37].

This study aligns with Bandura’s theory of reciprocal determinism, which posits that behavior, cognitive processes, and environmental factors interact in a bidirectional manner to influence each other [39]. This theory substantiates the interconnectedness and mediation effect of sense of school belonging, thereby providing a comprehensive framework for understanding their interrelationships. Teacher feedback, a critical educational environmental factor, significantly influences medical students’ self-perception and emotional well-being. When teacher feedback is fair and prompt, it can lead to positive emotional experiences such as self-satisfaction and pride [36]. These positive emotions not only contribute to an enhanced sense of happiness and fulfillment among medical students but also encourage their intrinsic motivation to engage in school activities [34]. This engagement, in turn, reinforces their sense of belonging and identification with the school [35]. This personal sense of belonging is closely linked to and positively influences self-regulated learning. A robust sense of school belonging fosters a positive emotional state among medical students [37] and significantly increases their learning motivation and self-efficacy [63]. When medical students feel a strong connection to their school, they are more likely to approach learning as a means of personal growth and self-improvement, thus exhibiting higher levels of self-regulated learning.

### Serial mediation roles of teacher-student interaction and sense of school belonging

This study reveals significant serial mediation effects of teacher-student interaction and sense of school belonging on the relationship between teacher feedback and medical students’ self-regulated learning, validating Hypothesis 4. Importantly, some scholars have reported that teacher-student interaction does not promote the sense of school belonging among public high school students [64]. This contrasts with the findings of the current study, possibly due to differences in the academic tasks and educational environments faced at various stages of education. Compared with those in high school, the contents of teacher-student interaction at the undergraduate level are more diverse, encompassing both course assignments and academic discussions as well as incorporating expanded content such as career guidance and scholarships [65]. With the increase in interaction content, medical students have a stronger demand for teacher-student interaction, which increases the likelihood of deriving a sense of school belonging from such interactions.

According to Bronfenbrenner’s ecological systems theory [15], the impact of teacher feedback on medical students’ self-regulated learning, which is mediated by teacher-student interaction and school belonging, can be understood through multiple environmental systems. Teacher feedback serves as a microsystemic influence that directly affects medical students’ self-regulated learning [27]. Additionally, it indirectly enhances self-regulated learning by fostering high-quality teacher-student interactions. These interactions, characterized by mutual respect, clear communication, and constructive dialog, create an environment conducive to learning and personal growth [29]. In turn, such positive teacher-student interactions serve as a potent catalyst for developing a strong sense of school belonging among medical students. The sense of school belonging, operating as a mesosystemic factor, reinforces students’ self-regulated learning by fostering a sense of connection, acceptance, and belonging within the school community [34]. This sense of belonging encourages students to engage more actively in their learning, take ownership of their education, and persist in the face of challenges. Therefore, a supportive educational environment that emphasizes positive teacher-student interaction and a strong sense of school belonging is crucial for fostering self-regulated learning among medical students.

*Contributions and implications.*

The research findings elucidate the intricate relationship between teacher feedback and the self-regulated learning of medical students, substantiating the individual and serial mediation effects of teacher-student interaction and sense of school belonging. These results offer insights into strategies aimed at cultivating highly competent healthcare professionals by fostering their self-regulated learning. First, teachers should integrate formative assessment into their teaching practices to promptly evaluate learning progress and address emerging challenges. This approach facilitates the provision of timely feedback, enabling teachers to refine their pedagogical strategies and effectively steer students’ learning endeavors. Second, teachers should enhance their interactions with medical students to address their academic, mental health, and career development concerns comprehensively. Through the adoption of a multifaceted communication strategy, teachers can provide essential support and foster an environment conducive to learning and personal growth. Finally, medical schools should establish a student-centered learning environment that values and acknowledges students’ contributions. By implementing a robust feedback mechanism, students can actively engage in school affairs, thereby augmenting their sense of belonging and empowering them throughout their educational journey.

### Limitations and prospects

This study has several limitations that warrant future exploration. First, although the study reveals variable correlations, the cross-sectional design employed limits the possibility of drawing causal relationships between variables. Future research might benefit from a longitudinal or quasi-experimental design to uncover the complex interplay between teacher feedback and other variables. Second, the use of self-report questionnaire may introduce measurement bias due to social desirability effects. Subsequent studies could employ multi-rater assessments, including evaluations from medical educators and administrators, and gather data through qualitative methods such as interviews and observations to obtain a more comprehensive and deeper understanding. Finally, the sample was drawn from a highly selective research university in China, where medical students are typically high achievers, which may limit the generalizability of the findings. To validate and compare the results, future studies should expand the sample to include medical colleges from various regions and with different levels of prestige.

## Data Availability

The data that support the findings of this study are available from H University but restrictions apply to the availability of these data, which were used under license for the current study, and so are not publicly available. Data are however available from the corresponding author upon reasonable request.
